# Uterine arteriovenous fistula: A case report and literature review

**DOI:** 10.1097/MD.0000000000043549

**Published:** 2025-08-01

**Authors:** Siyu Chen, Xia Song, Jinzhan Su, Fang Wu, Dongying Su, Shufeng Fan, Hui Hu, Jie Li, Miaoer Li, Jianmin Wen

**Affiliations:** a Graduate School of Zhejiang Chinese Medical University, Hangzhou, Zhejiang Province, China; b Radiology Department of Wuchang Street Community Health Service Center, Hangzhou, Zhejiang Province, China; c Radiology Department of the Second Affiliated Hospital of Zhejiang Chinese Medical University, Hangzhou, Zhejiang Province, China.

**Keywords:** angiography, arteriovenous fistula, case report, CT, CTA, diagnosis, DSA, imaging, MRI, uterine

## Abstract

**Rationale:**

Uterine arteriovenous fistula (UAVF) is not very common and can occur after traumatic activities, such as surgery, delivery, miscarriage, and curettage. Previous studies have reported on the ultrasound and digital subtraction angiography (DSA) findings of UAVF; however, there are few reports on computed tomography (CT), CT angiography (CTA), and magnetic resonance imaging (MRI) findings. We report a case in which residual products of conception were diagnosed during an MRI, with an oversight of the presence of UAVF.

**Patient concerns:**

A female aged 34 years presented with intermittent vaginal bleeding for 15 days that had increased in the last 2 hours, 43 days after an abortion. Ultrasound showed abnormal echo masses in the right uterine horn. MRI examination showed incomplete pregnancy. CT, CTA, and DSA examinations showed incomplete pregnancy with UAVF.

**Diagnoses:**

The postoperative combined pathological diagnosis was right uterine horn insufficiency abortion with UAVF.

**Interventions:**

To reduce intraoperative bleeding, interventional embolization was performed, followed by surgical resection.

**Outcomes:**

Recovery was good, and the patient was discharged.

**Lessons:**

After an induced abortion, especially after multiple abortions, in patients presenting with abdominal pain, irregular vaginal bleeding, sudden heavy bleeding, and other phenomena, we should consider the possibility of UAVF. In this paper, we report using CT, CTA, MRI, and DSA imaging findings of UAVF to improve our understanding of UAVF imaging performance. For patients with persistent bleeding and fertility requirements that require immediate surgical resection of the lesion, interventional embolization and resection of the lesion can be selected to reduce the risk of intraoperative bleeding and recurrence.

## 1. Introduction

Uterine arteriovenous fistula (UAVF), an uncommon disorder, can be divided into congenital, related to congenital vascular dysplasia, and acquired UAVF, which is related to trauma, infection, tumor, and other pathogeneses. Acquired UAVF occurs mostly secondary to a traumatic diagnosis and treatment activities (surgery, abortion, childbirth, and curettage)^[1]^ and is often manifested as irregular vaginal bleeding, sudden bleeding, and other phenomena.

This report details a case of UAVF, which was diagnosed with an incomplete pregnancy but overlooked the arteriovenous fistula. We discussed the clinical manifestations, imaging features, diagnosis, and treatment and conducted a literature review. This report aims to raise awareness among radiologists of the computed tomography (CT), CT angiography (CTA), and magnetic resonance imaging (MRI) manifestations of UAVF.

## 2. Case description

A female aged 34 years presented with intermittent vaginal bleeding for 15 days that had increased in the last 2 hours. The patient underwent an abortion in an outer hospital 43 days ago (the details are unknown). Vaginal bleeding occurred for 8 days after the abortion and then started again 15 days ago without obvious inducement. The volume was less than the normal menstrual flow, and bleeding occurred for 4 days. Furthermore, a small amount of coffee-colored discharge was found in the vagina intermittently. Two days before presenting to our hospital, the patient experienced abdominal distension, which was then relieved. However, 2 hours before the presentation, the patient experienced vaginal bleeding with no obvious cause, which consisted of a large volume (400–500 mL), was red in color with large blood clots, no abdominal pain and bloating, no dizziness, and no palpitation or other discomforts.

**Figure 1.**
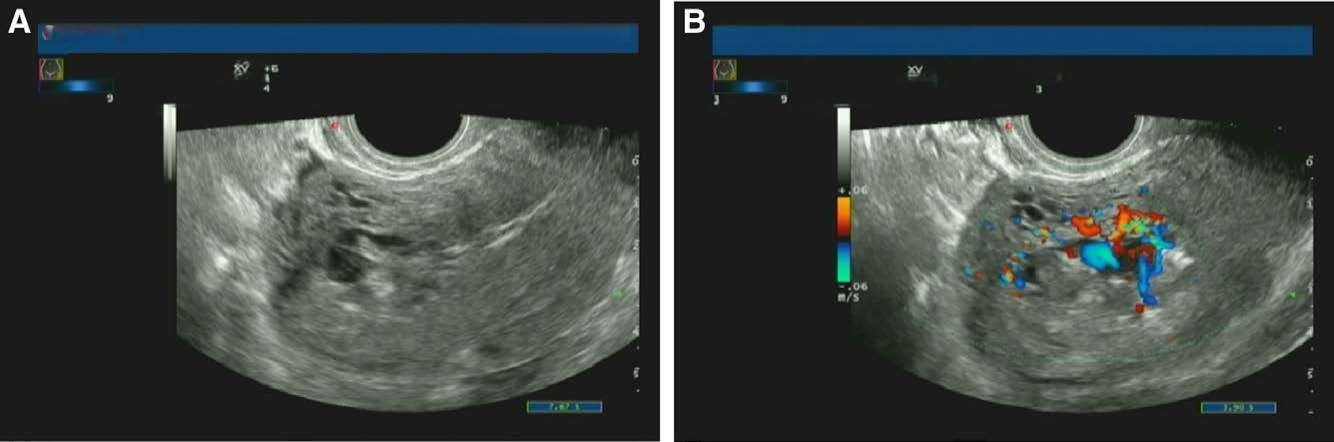
The ultrasound image shows uterine arteriovenous fistula (UAVF). The ultrasonic image showed an inhomogeneous echo mass at the right side of the uterine horn, about 3.5 × 3.1 cm in size, with unclear boundary and uneven internal echo. Many tortuous and dilated vascular echoes could be seen inside (A), and arterial and venous blood flow signals could be detected (B).

A urine examination showed that she was HCG-negative (20 mIU/mL). An outpatient color ultrasound revealed a heterogeneous echophore with tortuous dilation of the blood vessels (3.5 × 3.1 cm) at the right uterine horn, and the thickness of the endometrium was 0.47 cm (Fig. 1). There were no obvious abnormalities in the routine blood examination. After receiving symptomatic treatment, such as tranexamic acid 0.5 intravenous drip hemostasis and normal saline rehydration, the patient’s vaginal bleeding was significantly reduced, with slight abdominal pain, slight dizziness, no syncope, and no sweating or other discomforts.

**Figure 2.**
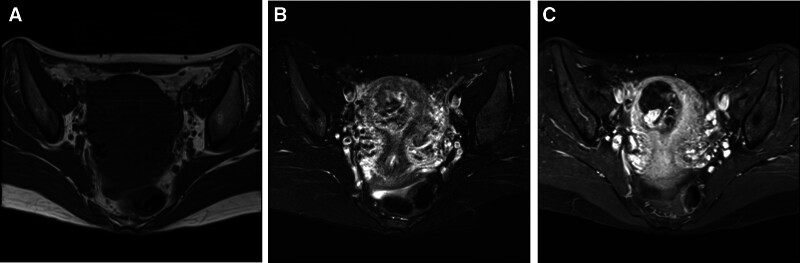
Magnetic resonance imaging (MRI) showing uterine arteriovenous fistula (UAVF). Axial T1-weighted MRI (A) shows a lesion in the right uterine angle and mixed isohyperintensity with a few high signals in it. Axial T2-weighted MRI (B) shows the lesion has mixed hyperintensity and “honeycomb sign” can be seen. Axial T1-weighted enhanced MRI (C) shows that the lesion is cystic. The cystic component is not enhanced, and the solid component is significant strengthening.

**Figure 3.**
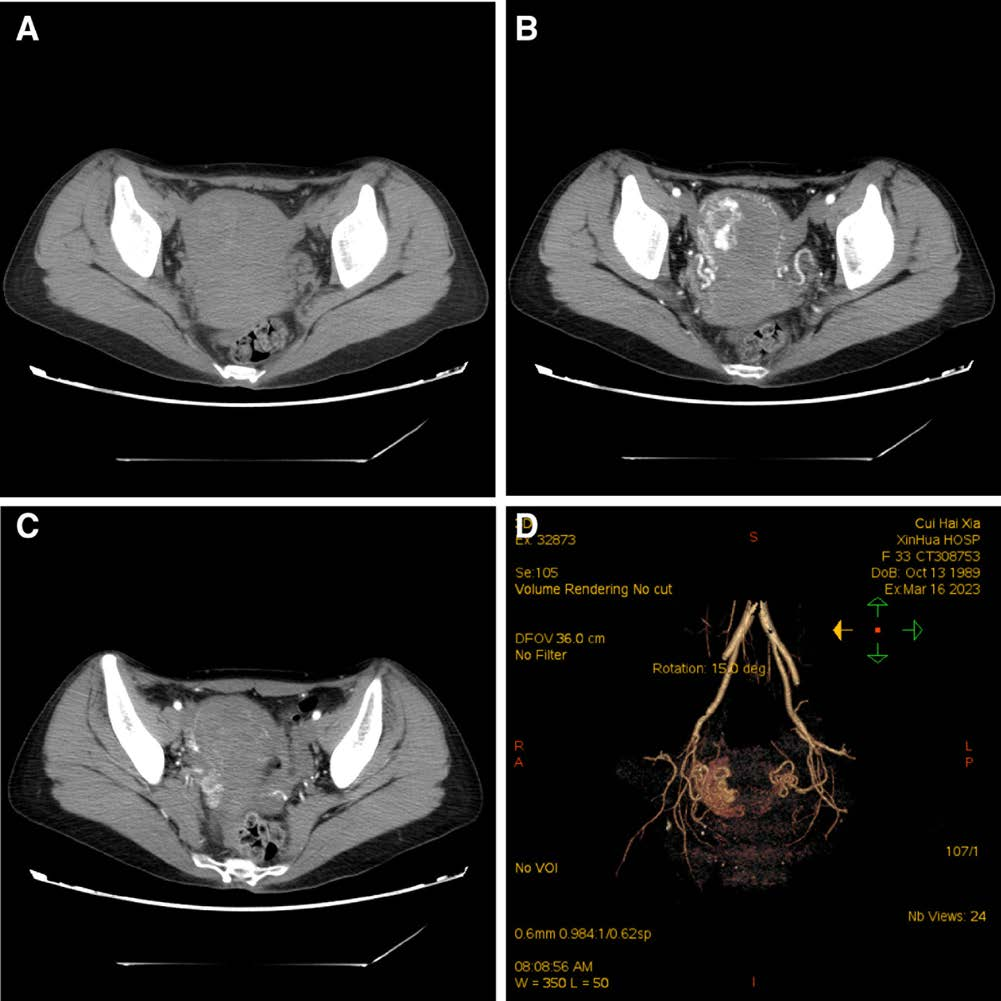
Computed tomography (CT) and CT angiography (CTA) images show uterine arteriovenous fistula (UAVF). Plain CT scan (A) shows that the lesion was located in the right uterine horn, and patchy hyperdensity was seen within it. An enhanced CT image (B) shows uneven and markedly enhanced lesions, similar to vascular enhancement. An enhanced CT image (C) shows that the right parametrial vein appears earlier than the contralateral side. CTA reconstruction (D) shows increased thickening and tortuosity of the right uterine artery branches, and multiple peripheral draining veins have developed.

**Figure 4.**
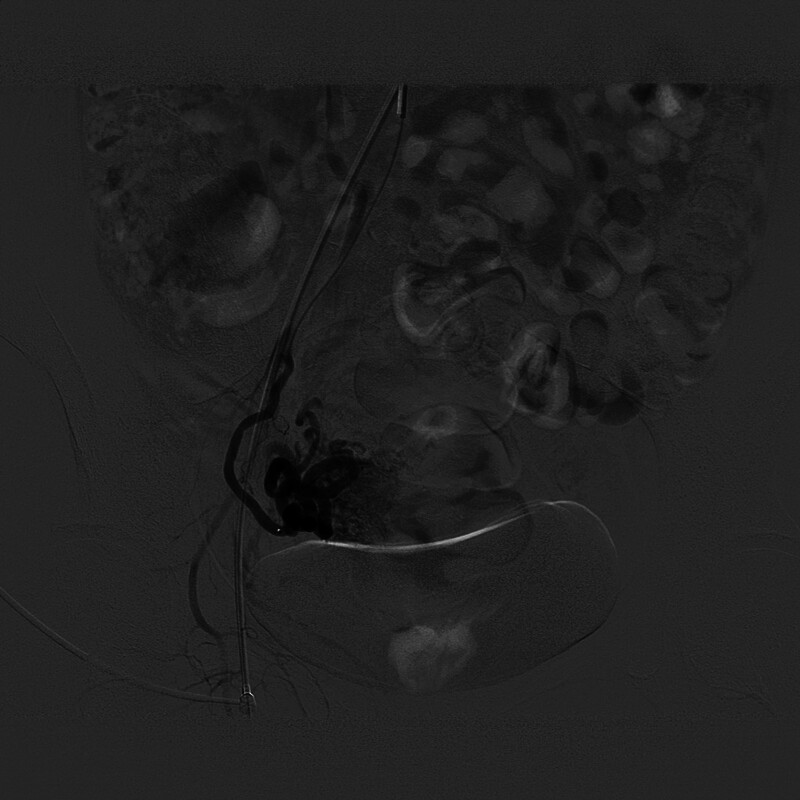
Digital subtraction. A DSA image shows uterine arteriovenous fistula (UAVF). The right uterine artery is markedly thickened and tortuous. Staining is visible in the right uterine cavity near the uterine angle, and some contrast media overflows. DSA = digital subtraction angiography.

The postadmission MRI examination revealed abnormal signals within the uterine cavity. T1-weighted MRI (T1WI) indicated a lesion at the right uterine angle with mixed isohyperintensity and a few high signals. T2-weighted MRI displayed that the lesion had mixed hyperintensity, and a “honeycomb sign” could be seen. Following contrast enhancement, T1WI demonstrated that the cystic component of the lesion did not enhance, while the solid component showed uneven significant strengthening (Fig. 2). Pregnancy in special locations, incomplete abortion, and gestational trophoblastic disease do not present with specific manifestations on MRI. They need to be differentiated from malignant trophoblastic diseases. Incomplete abortion of cornual pregnancy exhibits mixed signals on MRI images and has a high misdiagnosis rate. The lesion appears hypointense or isointense on T1WI, sometimes with mixed patchy hyperintense signals. On T2-weighted MRI, it shows a ring sign or honeycomb sign, and exhibits uneven significant enhancement on contrast scans. The lesions of the latter often have irregular borders, appearing serrated or moth-eaten with interruptions, showing significant enhancement in the early phase and exhibiting a rapid wash-in and wash-out pattern. Combined with the patient’s clinical history, the possibility of a residual pregnancy was considered, and further investigation was recommended. Since the first phase of enhancement was already the venous phase, the arteriovenous fistula around the lesion was missed. Plain CT revealed that the lesion was located in the right uterine horn, where patchy hyperdensities were observed. Enhanced CT demonstrated uneven and significantly enhanced lesions, resembling vascular enhancement, with the right parametrial vein appearing earlier than the contralateral side. CTA reconstruction indicated increased thickening and tortuosity of the right uterine artery branches, along with the development of multiple peripheral draining veins (Fig. 3). The solid component of the lesion exhibits high enhancement amplitude, with marked vascular-like enhancement, which aids in differentiation from other lesions. Based on the magnetic resonance findings, CT and CTA indicated a right uterine horn insufficiency abortion with UAVF. The patient was administered Rochefen 2.0 g qd intravenous drip anti-infective therapy, estrogen and progesterone artificial cycling, and mifepristone oral embryocidal treatment. The patient had no fertility requirements but was relatively young and wished to preserve the uterus. To reduce intraoperative blood loss during hysteroscopic surgery, hysterography and interventional embolization were performed before surgery. Contraindications were ruled out, and hysteroscopic uterine horn pregnancy removal and intrauterine adhesiolysis were performed after hysterography and embolization. Following uterine artery embolization, right internal iliac artery angiography showed that the right uterine artery was significantly thickened and tortuous. Staining could be seen in the right uterine cavity near the uterine angle, and some contrast agents overflowed to the bilateral uterine artery. After the contrast was confirmed, 300 to 500 µm PVA particles and a gelatin sponge were used to embolize the bilateral uterine artery. Postoperative angiography showed that the staining had disappeared, and there was no obvious contrast agent overflow (Fig. 4).

### Hysteroscopic surgery

The cervical canal was in a barrel, a large number of organic tissues occupied the right uterine cavity and uterine horn, and a large number of organic tissues were clamped and scraped out using oval forceps under ultrasound guidance. Furthermore, the organized tissue invaded the anterior wall and the muscularis of the uterine fundus, the peripheral blood vessels were abundant, and the right uterine horn was deep and distended. Ultrasound showed that the uterine wall was thin, and the right uterine wall had membranous adhesions. The residual organic tissue was resected to the superficial muscle layer, the right uterine wall adhesion tissue was separated, and local electrocoagulation was used to stop the bleeding.

### Conventional pathology

**Figure 5.**
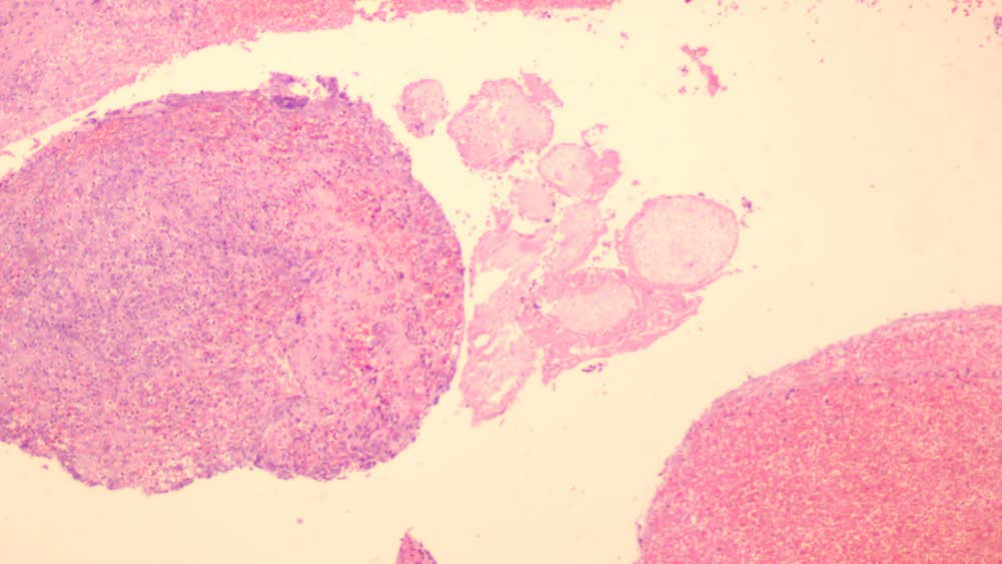
Microscopic examination reveals coagulated blood and degenerated necrotic material, with a small amount of degenerated villous tissue observed.

Microscopic examination reveals coagulated blood and degenerated necrotic material, with a small amount of degenerated villous tissue observed (Fig. 5). Postoperatively, Rochefen 2.0 g qd intravenous drip anti-infection symptomatic treatment was administered in addition to the continuation of the estrogen and progesterone artificial cycle. Follow-up with an ordinary color ultrasound showed no obvious abnormalities in the right lower abdomen. The patient recovered well and was discharged.

A telephone follow-up conducted one and a half months postoperatively revealed that the patient reported no discomfort such as vaginal bleeding or abdominal pain.

## 3. Discussion

UAVF after an induced abortion is caused by the presence of abnormal channels in uterine arteriovenous communication due to the absence of normal capillary network connections between the arteriovenous veins of the uterus and the direct shunt through the abnormal arteriovenous anastomosis, with an incidence of approximately 4.5%.^[2]^ There are no symptoms when the blood vessel is not ruptured; however, depending on the rupture’s location, different clinical symptoms can occur following blood vessel rupture. Communication with the abdominal cavity manifests as an intra-abdominal hemorrhage, and lower abdominal pain often occurs due to blood irritation of the peritoneum.^[3]^ Upon communication with the uterine cavity, vaginal bleeding occurs, often manifesting as irregular vaginal bleeding and sudden heavy bleeding. This is easily misdiagnosed by clinicians as other irregular vaginal bleeding diseases, resulting in an increased difficulty in the clinical diagnosis of UAVF.^[4]^ Previously, it was difficult to diagnose UAVF before surgery; the final diagnosis was mostly obtained through surgery and pathological diagnosis.^[5–7]^ Therefore, accurate preoperative diagnosis of UAVF remains a problem that still needs to be solved in clinical practice.

Previous imaging studies of UAVFs have commonly used ultrasound and uterine artery DSA. Ultrasonography is easily affected by the operator’s experience and the patient’s position, and when the lesion is deep or close to the uterine margin, it is often missed. There are also limitations to ultrasonography: the acoustic window is narrow, and the arteries supplying the UAVF, the veins draining, and the possible collateral vessels are poorly displayed.^[8]^ Moreover, the widespread use of ultrasonography has increased the false-positive rate of UAVF diagnosis.^[9]^ At present, qualitative diagnosis of UAVF has been proposed by scholars at home and abroad, and the display of blood supply arteries, drainage veins, and malformed blood vessels is the key to confirming the diagnosis of UAVF. DSA is the “gold standard” for diagnosing UAVF, which helps to determine whether there is abnormal arteriovenous communication. With an intraoperative diagnosis, uterine arterial embolization can be performed at the same time, and the feeding artery can be quickly identified, the fistula embolized, and the bleeding stopped.^[10]^ However, DSA requires a high level of equipment configuration and skill level of the operator, and it is an invasive test with associated radiation damage; therefore, it is not recommended to perform hysterography early in patients with suspected UAVF.^[1]^

Previously, CT, CTA, MRI, and magnetic resonance angiography (MRA) manifestations of UAVF have rarely been reported. CT or MRI can determine the morphology, location, and adjacent tissue involvement of the uterus and malformed vessels. CTA technology is becoming increasingly mature, and it has the advantages of high spatial resolution, large scanning range, and rapid vascular imaging with only intravenous contrast medium, which can clearly show the location, size, and spatial relationship of the common iliac artery, internal and external iliac artery, and their branches, and the uterine artery and its vascular mass.

Typical MRI shows patchy mixed signal shadows in the uterus, with unclear borders and multiple vascularities of varying thickness and increased tortuosity.^[11]^ On MRI-enhanced scans, UVAF is characterized by a network of thickened supplying arteries communicating directly with the draining veins, which can be visualized during the arterial phase.^[12]^ MRA can provide more information about the size and location of malformed blood vessels, as well as the arteries and drainage veins that supply blood vessels, and can also be used to observe the relationship between lesions and the adnexa and endometrium in detail.^[13]^ This case retrospectively analyzes the reason for the MRI missing the diagnosis of a UAVF, which was the absence of MRA images, failing to provide relevant information about the feeding artery and draining vein.

Treatment of acquired UAVF includes conservative treatment, hysterectomy, lesion resection, and interventional embolization. Conservative treatment includes drug therapy and local compression to stop the bleeding; however, these only achieve temporary hemostasis. Hysterectomy is mainly performed in patients with recurrent uterine bleeding who have no fertility requirements; however, it is traumatic, has many complications, and can induce great physical and psychological trauma to the patient.^[14]^ Lesion resection is primarily aimed at women who wish to preserve their fertility or patients who want to maintain their uterus, such as those undergoing hysteroscopic UAVF lesion resection. However, it is only suitable for patients who are hemodynamically stable without bleeding. Intrauterine manipulation is not recommended for patients with a definite diagnosis of UAVF with vaginal bleeding, as it may damage the malformed vessel mass and cause massive bleeding.^[15]^ Interventional embolization is a nonsurgical method with a significant effect of preserving the uterus. Selective uterine artery embolization can quickly and directly cut off the blood supply to the bleeding site to achieve timely hemostasis. Timor et al^[16]^ suggested that uterine artery embolization does not eliminate large vascular lakes and that refractory vaginal bleeding can still occur.

In this case, considering the patient’s persistent bleeding symptoms and fertility requirements, hysteroscopic resection of the lesion after preoperative interventional embolization was used, which reduced the risk of intraoperative bleeding and reduced recurrence to meet the requirements of radical cure.

## Acknowledgments

The authors thank the patient and his family for giving permission for his inclusion in this study. The authors thank the family for their support.

## Author contributions

**Conceptualization:** Siyu Chen, Miaoer Li, Jianmin Wen.

**Writing – original draft:** Siyu Chen, Xia Song, Miaoer Li.

**Writing – review & editing:** Jinzhan Su, Shufeng Fan, Miaoer Li.

**Data curation:** Fang Wu, Dongying Su, Hui Hu, Jie Li, Miaoer Li.

**Supervision:** Shufeng Fan, Miaoer Li.
